# Explainable machine learning for hypertension prevalence classification: a cross-sectional study in Hainan Province, China

**DOI:** 10.3389/fcvm.2026.1871561

**Published:** 2026-07-17

**Authors:** Yuewei Wu, Shan Huang, Miaomiao Qi, Yuanyuan Zhang, Mei Lu, Tianfa Li, Yueqiong Kong

**Affiliations:** Key Laboratory of Emergency and Trauma of Ministry of Education, Department of Cardiology, The First Affiliated Hospital, Hainan Medical University, Haikou, China

**Keywords:** cardiovascular diseases, China, explainability, hypertension, machine learning

## Abstract

**Objective:**

This study used explainable machine learning models to classify prevalent hypertension status in the general population.

**Methods:**

This cross-sectional study used clinical data collected with questionnaires, physical examinations, blood biochemistry, and routine urine tests from 4,800 permanent residents aged ≥18 years old in Hainan Province, China, from 2021 to 2022. The random forest algorithm was applied to select the most significant features based on importance scores of all variable features. Models for hypertension prevalence classification were created using six machine learning techniques. These models were then compared to select a model with high classification performance based on classification accuracy and the area under curve (AUC) values. Calibration assessment and decision curve analysis were additionally performed to assess clinical applicability. To evaluate and illustrate the best models, the SHapley Additive Explanation (SHAP) values and the Local Interpretable Model-Agnostic Explanations (LIME) algorithms were used.

**Results:**

In total, 4,606 permanent residents were included in this study (hypertension, 32.5%). They were randomly split into two groups: a training set (3,224, 70%) and a validation set (1,382, 30%). After the random forest algorithm was applied to score feature importance, the top 10 most important features, including age, smoking, urine microalbumin (UALB), educational level, diabetes, body mass index (BMI), sex, triglyceride (TG), income level, and family history of hypertension, were finally included for model construction. With an AUC of 0.8461, the eXtreme Gradient Boosting (XGBoost) model had the greatest performance. The SHAP values were used to quantify the contribution of each input feature to the XGBoost model and demonstrate the importance ranking of predictors. The LIME algorithm integrated the SHAP values to provide a more compelling explanation for each individual classification result. A web page based on the results of this study was developed to support hypertension prevalence screening in clinical environments.

**Conclusion:**

A machine learning model based on clinical variables was developed and verified, which showed superior performance in identifying prevalent hypertension cases in the general population, opening up new possibilities for the rapid population screening and prevention of hypertension.

## 1 Introduction

Hypertension is one of the most critical risk factors for cardiovascular disease, stroke, and kidney disease (1). Hypertension is one of the most significant public health problems worldwide, which, according to the World Health Organization, accounts for nearly 8.5 million deaths globally (2). Meanwhile, prevalence of hypertension doubled from 1990 to 2019 and it is expected that 1.56 billion will be hypertensive worldwide by 2025 (3, 4). In China, about 23.2% of the population had hypertension in 2015, about 245 million people have this disease, and its prevalence is expected to rise even further (5). Low rates of hypertension awareness, diagnosis, treatment, and management exist, particularly in less developed areas account for this rapid increase in prevalence (6). Therefore, it is crucial to enhance hypertension screening in the general population and provide preventive intervention and treatment for high-risk people. Although much work and effort have been made to prevent and treat hypertension, presently, there is no workable solution for considerably lessening the health burden of hypertension in the general population.

Early identification of individuals with undiagnosed prevalent hypertension will help inform awareness programs and optimize the allocation of limited medical resources to effectively reduce the prevalence of hypertension. In recent years, machine learning (ML) has emerged as an effective technique for population-specific prevalence classification (7–9). Among the advantageous features of machine learning methods is the ability to process large-scale data and uncover correlations between data to classify disease status (10). This can significantly improve disease diagnosis reliability, performance, and accuracy (11). Although several machine learning-based models for hypertension prevalence screening have been created, the “black-box” nature of the algorithms require that clinicians understand the machine learning decision logic during the application to achieve accurate and safe classification outputs (12–16). Previously established models for hypertension screening are based on traditional statistical techniques, which limits their performance and application in the clinical setting.

Conventional black-box machine learning models lack transparent decision pathways, which restricts clinical translation. Explainable machine learning techniques are specifically designed to unpack internal model logic, enabling clinicians to trace feature contributions and fully comprehend the reasoning behind classification outputs (17, 18). SHapley Additive Explanation (SHAP) and Local Interpretable Model-Agnostic Explanations (LIME) are the two most popular interpretable approaches (19, 20). Machine learning models based on these explainable methods have been applied to explain mortality in heart failure patients and to classify the first acute exacerbation in patients with chronic obstructive pulmonary disease and the occurrence of postoperative malnutrition in children with congenital heart disease (21–23). The explainable analysis methods provide reasonable explanations for the results of these classification models, supporting their application in clinical settings. In this study, we applied machine learning methods to classify prevalent hypertension and identify undiagnosed community patients. In addition, we used explainable machine learning methods to provide clinicians with black-box explainability.

## 2 Methods

### 2.1 Data source and study population

All participant data used in this study were obtained from the “Cardiovascular Disease Surveillance and Risk Factors in the Chinese Population” project. From July 2021 to January 2022, four cities and counties in Hainan Province were selected using multilayer multi-stage random sampling and participants were chosen according to the probability sampling method proportional to the population size. Using simple random sampling, four townships were selected in the selected cities and counties and three resident committees were randomly selected in each township. Each resident committee was divided into 14 layers according to gender and age groups from which a corresponding number of individuals was chosen using simple random sampling method. Subsequently, 1,200 people were selected in each of the four cities and counties, resulting in a total sample of 4,800 people. However, 194 people who did not complete the research or whose data were lost were excluded. Finally, 4,606 participants were enrolled in this study.

### 2.2 Data collection

The survey comprised a face-to-face questionnaire, physical examination, and laboratory tests. Qualified investigators trained by the provincial Centers for Disease Control and Prevention and hospitals using electronic tablets collected basic information from respondents through face-to-face interviews, including lifestyle, dietary characteristics, disease history, and family history. The physical examination included height, weight, and blood pressure measurements. Laboratory tests included four lipid panels [triglyceride (TG), high-density lipoprotein (HDL), low-density lipoprotein (LDL), and total cholesterol (TC)], uric acid (UA), serum creatinine (SCR), and urinary albumin (UALB).

### 2.3 Definitions

Hypertension was defined as having a mean systolic blood pressure ≥ 140 mmHg and/or a mean diastolic blood pressure ≥ 90 mmHg, a previous diagnosis of hypertension, or currently taking anti-hypertensive medication (24). Dyslipidemia: TG ≥ 2.26 mmol/L, TC ≥ 6.22 mmol/L, LDL ≥ 4.14 mmol/L, and HDL < 1.04 mmol/L. BMI was calculated as the ratio of weight to the square of height, which was then classified into four categories: underweight (<18.5 kg/m^2^), normal (18.5–23.9 kg/m^2^), overweight (24.0–27.9 kg/m^2^), and obese (≥28.0 kg/m^2^). Participants with UA level ≥ 420 μmol/L were deemed to have high uric acid. High serum creatinine: SCR > 133 μmol/L (male), SCR > 106 μmol/L (female). High urine microalbumin: UALB > 20 μg/mL.

### 2.4 Statistical analysis

To align with the routine workflow of grassroots population hypertension screening, improve clinical interpretability of the model, and enhance robustness against real-world survey data noise, all continuous clinical indicators (including age, BMI, and triglycerides) were discretized into categorical variables in this study. All stratification cut-offs were strictly derived from authoritative Chinese clinical guidelines and standardized epidemiological survey protocols, rather than arbitrarily defined by researchers. Specifically, BMI stratification followed the Chinese Guidelines for the Prevention and Control of Overweight and Obesity in Adults; triglyceride classification adopted cut-offs from the Chinese Guidelines for the Management of Dyslipidemia in Adults; age grouping used standard stratification widely applied in domestic population-based cross-sectional cardiovascular studies. This guideline-based discretization minimizes artificial boundary bias from arbitrary cut-point setting. Meanwhile, categorical input matches the conventional data recording format of primary care public health archives, lowers the operational threshold of the supporting online screening tool, and reduces input errors for frontline medical staff during large-scale population screening. Although discretization leads to minor loss of fine-grained numerical information, this trade-off balances model stability and clinical practicality for population-level preliminary hypertension screening, which is the core positioning of this work. For categorical variables, chi-square tests were utilized to analyze differences between test groups represented as numbers (percentages). *P* values less than 0.05 (2-sided) were regarded as statistically significant for all statistical analyses, which were carried out using Python (version 3.8.3) and R software (version 4.1.3).

### 2.5 Machine learning model

All modeling procedures strictly followed the principle of avoiding data leakage: all preprocessing, feature screening and hyperparameter tuning steps are fitted exclusively on the training set, and the validation set only applied the fixed rules without secondary refitting. A unified fixed random seed was set for all stochastic procedures to ensure full experimental reproducibility. Before developing the model, we performed data exploration and feature screening. Briefly, all participants were randomly divided into a training set (70%) and a validation set (30%). Random forest feature importance ranking and feature filtering were implemented only within the training cohort after sample splitting (25). No validation data was involved in feature evaluation, which effectively prevents data leakage bias.

Classification models for identifying concurrent hypertension were developed and validated using six machine learning techniques: gradient boosting (GBDT), adaptive boosting (AdaBoost), decision trees (DT), *k*-nearest neighbors (KNN), logistic regression (LR), and extreme gradient boosting (XGBoost).

Class imbalance handling: Given the mild class imbalance (32.5% hypertension prevalence), the Synthetic Minority Oversampling Technique (SMOTE) was applied exclusively to the training dataset to generate synthetic samples for the minority class. The validation set retained its original distribution without any resampling.

Hyperparameter tuning: Grid search combined with 5-fold cross-validation was performed strictly within the training set, with AUC as the optimization target. The search ranges and final optimal parameters for all six models are summarized in Table 1.

**Table 1:** Hyperparameter search ranges and optimal values of six machine learning models.

ML model	Hyperparameter	Search range	Optimal value
GBDT	max_depth	6, 7, 8	8
	learning rate	0.3, 0.4, 0.5	0.4
	subsample	0.6, 0.8, 1.0	1.0
AdaBoost	n_estimators	1, 5, 10	10
	learning rate	0.01, 0.05, 0.1	0.1
	algorithm	SAMME, SAMME.R	SAMME.R
DT	max_depth	1, 2, 3	3
	min_samples_split	1, 5, 10	5
	min_samples_leaf	1, 2, 3	1
KNN	n_neighbors	3, 5, 7	7
	weights	uniform, distance	distance
	metric	euclidean, manhattan	manhattan
LR	solver	liblinear, lbfgs, newton-cg	liblinear
	class_weight	None, balanced	None
	tol	0.0001, 0.001, 0.01	0.0001
XGBoost	n_estimators	100, 300, 500	300
	max_depth	3, 4, 5	5
	learning_rate	0.07, 0.08, 0.09	0.09

ML, machine learning; GBDT, gradient boosting; AdaBoost, adaptive boosting; DT, decision tree; KNN, *k*-nearest neighbors; LR, logistic regression; XGBoost, eXtreme gradient boosting.

Classification threshold: The default threshold of 0.5 was adopted for all models, and classification accuracy was used as one of the core performance evaluation metrics.

**Figure 1:**
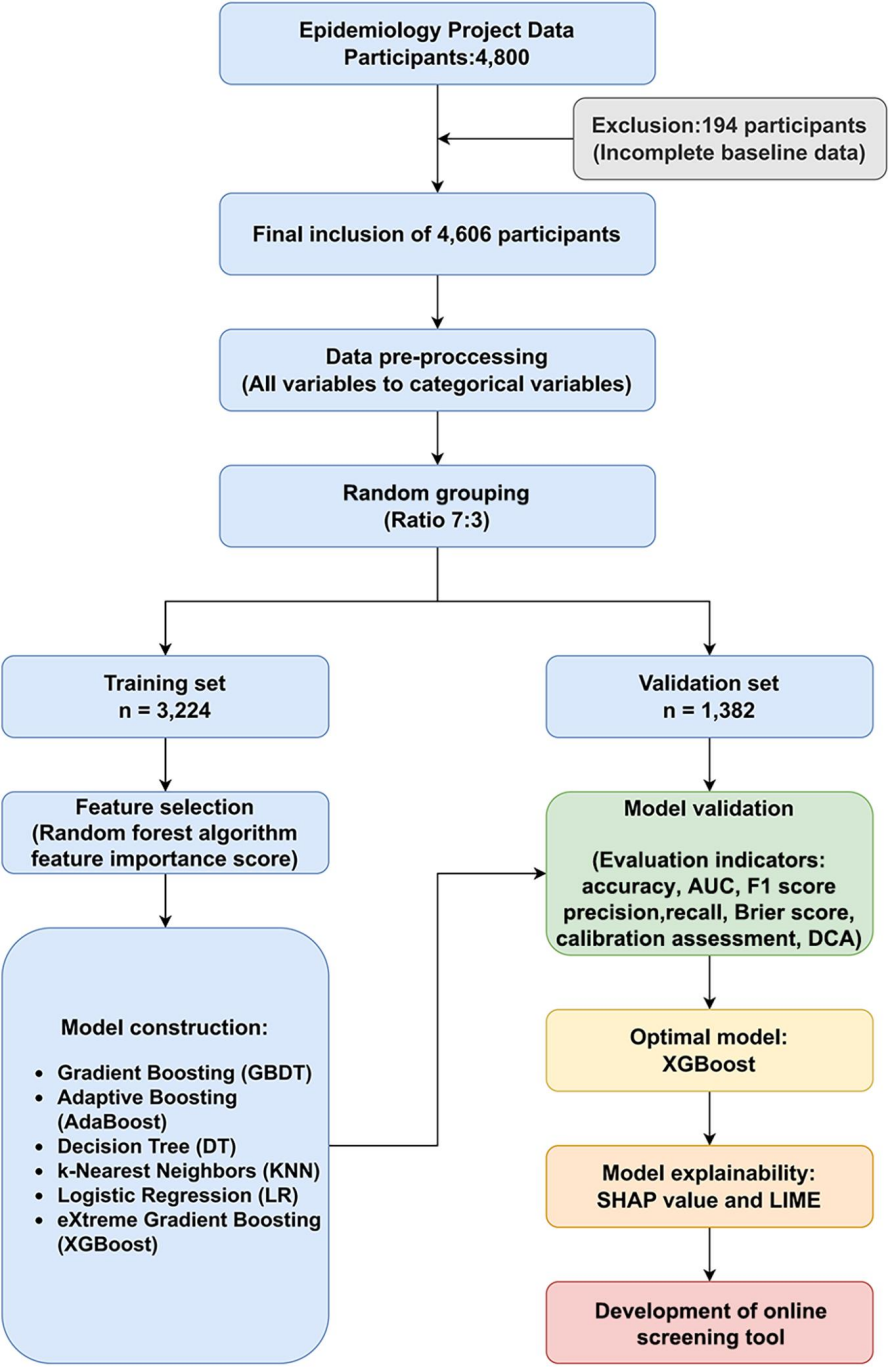
Flow chart of this study. AUC, the area under curve; DCA, decision curve analysis; SHAP, SHapley additive explanation; LIME, Local interpretable model-agnostic explanations.

Model performance was comprehensively evaluated from three dimensions: discrimination (AUC, accuracy, *F*1 score, precision, recall), calibration (calibration curves, Hosmer–Lemeshow test, Brier score) and clinical net benefit (decision curve analysis, DCA). Among them, the optimal model was selected based on comprehensive discriminative and calibration performance. We then visualized the important features affecting the risk of hypertension using the SHAP values in the optimal model, analyzed the importance of individual features affecting the model output, and visualized the impact of key features in the optimal model on individual samples jointly with the LIME algorithm. Finally, we developed an online web page based on Streamlit Python that allows users to select a machine learning model and input feature parameters to obtain classification results and estimated prevalence probabilities (26). Figure 1 shows a flow chart of this research design.

## 3 Results

### 3.1 Participants

Initially, 4,800 people were enrolled in this survey, but 194 respondents withdrew, leaving 4,606 people as the final sample. Of these, 1,497 individuals were hypertensive, with a crude prevalence rate of 32.5%. Compared with the non-hypertensive group, the hypertensive group was more likely to be associated with educational level, income level, alcohol consumption, fruit intake, meat intake, egg intake, fried food intake, activity time, sedentary time, sleep time, diabetes, family history of hypertension, family history of diabetes, family history of hyperlipidemia, BMI, TG, HDL, LDL, TC, UA, SCR and UALB. These associations are shown in Table 2. Furthermore, 2,108 (45.8%) of the total participants were male and 2,498 (54.2%) were female. Males were more likely to have hypertension than females—a prevalence rate of 40.4% vs. 25.9%. Further analysis of the differences in the prevalence of hypertension by age group revealed that hypertension was more prevalent in the 18–30, 31–40, 41–50, and 51–60 age groups for males and over 60 age group for females. Detailed results of prevalence analysis are shown in Table 3. The risk of having prevalent hypertension increased with age in both males and females. Meanwhile, compared with non-smokers, the prevalence of hypertension was higher among smokers. In total, there were 1,765 smokers (38.3%), of which males accounted for 1,394 (80%).

**Table 2:** Baseline data of participants in this study.

Features	Total	Non-hypertension	Hypertension	*P* value
	*N* = 4,606	*N* = 3,109	*N* = 1,497	
	*n* (%)	*n* (%)	*n* (%)	
Sex				<0.001
Female	2,498 (54.2%)	1,852 (59.6%)	646 (43.2%)	
Male	2,108 (45.8%)	1,257 (40.4%)	851 (56.8%)	
Age, years				<0.001
18–30	1,001 (21.7%)	936 (30.1%)	65 (4.4%)	
31–40	954 (20.7%)	792 (25.5%)	162 (10.8%)	
41–50	876 (19.0%)	606 (19.5%)	270 (18.0%)	
51–60	803 (17.4%)	417 (13.4%)	386 (25.8%)	
>60	972 (21.2%)	358 (11.5%)	614 (41.0%)	
Educational level				<0.001
Not educated	394 (8.6%)	167 (5.5%)	227 (15.2%)	
Primary school	826 (17.9%)	446 (14.3%)	380 (25.4%)	
Middle school	1,534 (33.3%)	1,018 (32.7%)	516 (34.4%)	
High school	858 (18.6%)	619 (19.9%)	239 (16.0%)	
College or above	994 (21.6%)	859 (27.6%)	135 (9.0%)	
Income level, thousand/year				<0.001
<10	1,425 (30.8%)	846 (27.2%)	579 (38.7%)	
10–20	1,268 (27.5%)	851 (27.4%)	417 (27.9%)	
21–30	749 (16.3%)	541 (17.4%)	208 (13.9%)	
31–50	585 (12.7%)	430 (13.8%)	155 (10.4%)	
51–100	445 (9.7%)	341 (11.0%)	104 (6.9%)	
>100	134 (3.0%)	100 (3.2%)	34 (2.2%)	
BMI, kg/m^2^				<0.001
Underweight	422 (9.2%)	347 (11.2%)	75 (5.0%)	
Normal	2,362 (51.3%)	1,732 (55.7%)	630 (42.1%)	
Overweight	1,383 (30.0%)	808 (26.0%)	575 (38.4%)	
Obesity	439 (9.5%)	222 (7.1%)	217 (14.5%)	
DM				<0.001
No	3,886 (84.4%)	2,783 (89.5%)	1,103 (73.7%)	
Yes	720 (15.6%)	326 (10.5%)	394 (26.3%)	
Alcohol use				<0.001
No	3,603 (78.2%)	2,497 (80.3%)	1,106 (73.9%)	
Yes	1,003 (21.8%)	612 (19.7%)	391 (26.1%)	
Smoking				<0.001
No	2,841 (61.7%)	2,198 (70.7%)	643 (43.0%)	
Yes	1,765 (38.3%)	911 (29.3%)	854 (57.0%)	
Drinking tea				0.115
No	2,334 (50.7%)	1,601 (51.5%)	733 (49.0%)	
Yes	2,272 (49.3%)	1,508 (48.5%)	764 (51.0%)	
Vegetables, >500 g/day				0.328
No	4,429 (96.2%)	2,996 (96.4%)	1,433 (95.7%)	
Yes	177 (3.8%)	113 (3.6%)	64 (4.3%)	
Fruits, >350 g/day				0.006
No	4,250 (92.3%)	2,845 (91.5%)	1,405 (93.9%)	
Yes	356 (7.7%)	264 (8.5%)	92 (6.1%)	
Meats, >75 g/day				<0.001
No	1,241 (26.9%)	736 (23.7%)	505 (33.7%)	
Yes	3,365 (73.1%)	2,373 (76.3%)	992 (66.3%)	
Aquatic products, >75 g/day				0.251
No	2,479 (53.8%)	1,692 (54.4%)	787 (52.6%)	
Yes	2,127 (46.2%)	1,417 (45.6%)	710 (47.4%)	
Eggs, >75 g/day				<0.001
No	3,681 (79.9%)	2,424 (78.0%)	1,257 (84.0%)	
Yes	925 (20.1%)	685 (22.0%)	240 (16.0%)	
Dairy products, >300 g/day				0.674
No	4,503 (97.8%)	3,037 (97.7%)	1,466 (97.9%)	
Yes	103 (2.2%)	72 (2.3%)	31 (2.1%)	
Fried food, >30 g/day				0.001
No	4,170 (90.5%)	2,784 (89.5%)	1,386 (92.6%)	
Yes	436 (9.5%)	325 (10.5%)	111 (7.4%)	
Activity time, h/day				<0.001
<2.5	823 (17.9%)	533 (17.1%)	290 (19.4%)	
2.5–5	680 (14.8%)	522 (16.8%)	158 (10.5%)	
>5	3,103 (67.3%)	2,054 (66.1%)	1,049 (70.1%)	
Sedentary time, h/day				<0.001
<4	2,559 (55.6%)	1,651 (53.1%)	908 (60.7%)	
4–8	1,513 (32.8%)	1,041 (33.5%)	472 (31.5%)	
>8	534 (11.6%)	417 (13.4%)	117 (7.8%)	
Sleep time, h/day				<0.001
<6	377 (8.1%)	213 (6.9%)	164 (11.0%)	
6–7.9	2,960 (64.3%)	2,024 (65.1%)	936 (62.5%)	
8–10	1,041 (22.6%)	727 (23.4%)	314 (21.0%)	
>10	228 (5.0%)	145 (4.6%)	83 (5.5%)	
Family history of hypertension				<0.001
No	3,602 (78.2%)	2,487 (80.0%)	1,115 (74.5%)	
Yes	1,004 (21.8%)	622 (20.0%)	382 (25.5%)	
Family history of DM				0.002
No	4,373 (94.9%)	2,930 (94.2%)	1,443 (96.4%)	
Yes	233 (5.1%)	179 (5.8%)	54 (3.6%)	
Family history of hyperlipidemia				0.02
No	4,485 (97.4%)	3,015 (97.0%)	1,470 (98.2%)	
Yes	121 (2.6%)	94 (3.0%)	27 (1.8%)	
Family history of CHD				0.487
No	4,489 (97.5%)	3,034 (97.6%)	1,455 (97.2%)	
Yes	117 (2.5%)	75 (2.4%)	42 (2.8%)	
Family history of stroke				0.135
No	4,489 (97.5%)	3,038 (97.7%)	1,451 (96.9%)	
Yes	117 (2.5%)	71 (2.3%)	46 (3.1%)	
High TG				<0.001
No	4,048 (87.9%)	2,847 (91.6%)	1,201 (80.2%)	
Yes	558 (12.1%)	262 (8.4%)	296 (19.8%)	
High TC				<0.001
No	3,923 (85.2%)	2,748 (88.4%)	1,175 (78.5%)	
Yes	683 (14.8%)	361 (11.6%)	322 (21.5%)	
Low HDL				<0.001
No	4,178 (90.7%)	2,857 (91.9%)	1,321 (88.2%)	
Yes	428 (9.3%)	252 (8.1%)	176 (11.8%)	
High LDL				<0.001
No	3,940 (85.5%)	2,719 (87.5%)	1,221 (81.6%)	
Yes	666 (14.5%)	390 (12.5%)	276 (18.4%)	
High SCR				<0.001
No	4,571 (99.2%)	3,101 (99.7%)	1,470 (98.2%)	
Yes	35 (0.8%)	8 (0.3%)	27 (1.8%)	
High UA				<0.001
No	3,559 (77.3%)	2,515 (80.9%)	1,044 (69.7%)	
Yes	1,047 (22.7%)	594 (19.1%)	453 (30.3%)	
High UALB				<0.001
No	3,263 (70.8%)	2,449 (78.8%)	814 (54.4%)	
Yes	1,343 (29.2%)	660 (21.2%)	683 (45.6%)	

BMI, body mass index; DM, diabetes mellitus; CHD, coronary heart disease; TG, triglyceride; TC, total cholesterol; HDL, high-density lipoprotein; LDL, low-density lipoprotein; SCR, serum creatinine; UA, uric acid; UALB, urinary albumin.

**Table 3:** Differences in the prevalence of hypertension by gender in each age group.

Features	Total	Non-hypertension	Hypertension	Prevalence	*P* value
	*n* (%)	*n* (%)	*n* (%)	%	
Sex					<0.001
Female	2,498 (54.2)	1,852 (59.6)	646 (43.2)	25.9	
Male	2,108 (45.8)	1,257 (40.4)	851 (56.8)	40.4	
Age group					
18–30					<0.001
Female	506 (50.5)	502 (53.6)	4 (6.2)	0.8	
Male	495 (49.5)	434 (46.4)	61 (93.8)	12.3	
31–40					<0.001
Female	551 (57.8)	513 (64.8)	38 (23.5)	6.9	
Male	403 (42.2)	279 (35.2)	124 (76.5)	30.8	
41–50					<0.001
Female	488 (55.7)	387 (63.9)	101 (48.8)	20.7	
Male	388 (44.3)	219 (36.1)	169 (51.2)	43.6	
51–60					<0.001
Female	438 (54.5)	264 (63.3)	174 (45.1)	39.7	
Male	365 (45.5)	153 (36.7)	212 (54.9)	58.1	
>60					0.624
Female	515 (53.0)	186 (52.0)	329 (53.6)	63.9	
Male	457(47.0)	172(48.0)	285(46.4)	62.4	

### 3.2 Predictor selection

**Figure 2:**
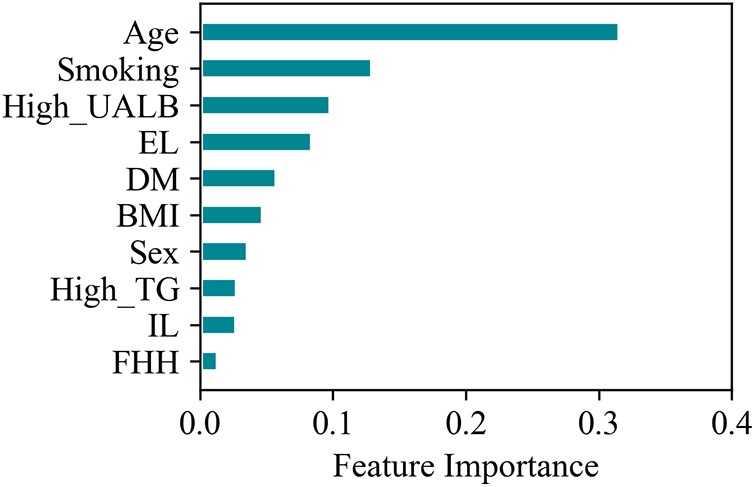
Feature selection was performed in the training set using the random forest algorithm feature importance score. BMI, body mass index; DM, diabetes mellitus; EL, educational level; FHH, family history of hypertension; IL, income level; TG, triglyceride; UALB, urinary albumin.

**Figure 3:**
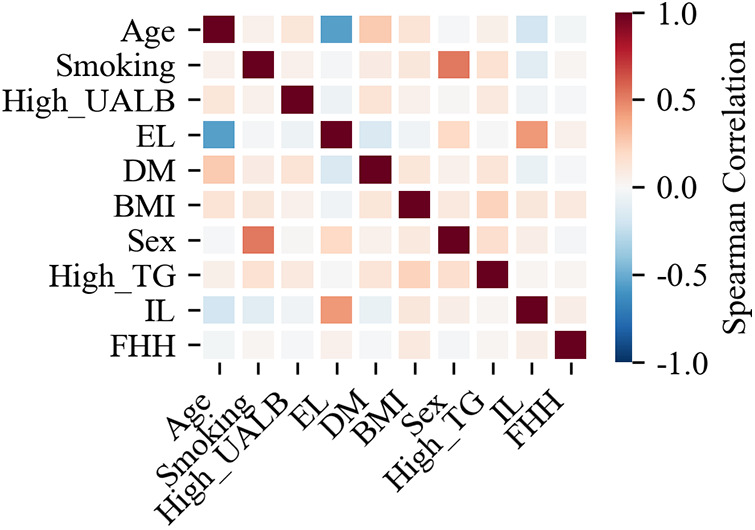
Correlation of the 10 key features. These features were independent of each other and there was no significant covariance. EL, educational level; IL, income level; BMI, body mass index; DM, diabetes mellitus; FHH, family history of hypertension; TG, triglyceride; UALB, urinary albumin.

A total of 31 features were included in this study. Among them, the top 10 key features were finally included in the random forest algorithm feature importance scoring. All feature importance calculations and filtering were performed exclusively within the training dataset after train-validation splitting, consistent with the workflow shown in Figure 1. Ranked in order of importance, these features were age, smoking, UALB, educational level, diabetes, BMI, sex, TG, income level, and family history of hypertension (Figure 2). Then, we assessed the correlations among these 10 key features in the heat map. As can be seen in Figure 3, the features are independent of each other and show no significant covariance.

### 3.3 Model development and validation

**Figure 4:**
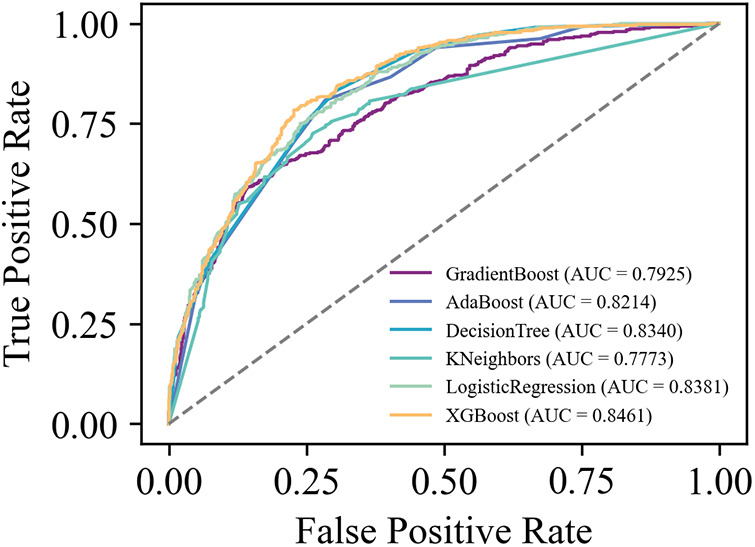
AUC of six machine learning models in the validation set. AUC, the area under curve; ROC, receiving operating characteristic curve. AdaBoost, adaptive boosting; XGBoost, eXtreme gradient boosting.

**Figure 5:**
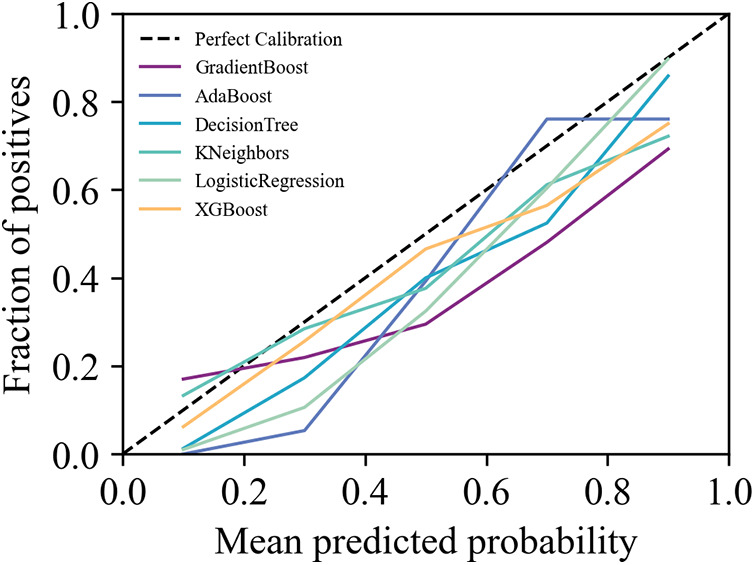
Calibration curve of six machine learning models. AdaBoost, adaptive boosting; XGBoost, eXtreme gradient boosting.

**Figure 6:**
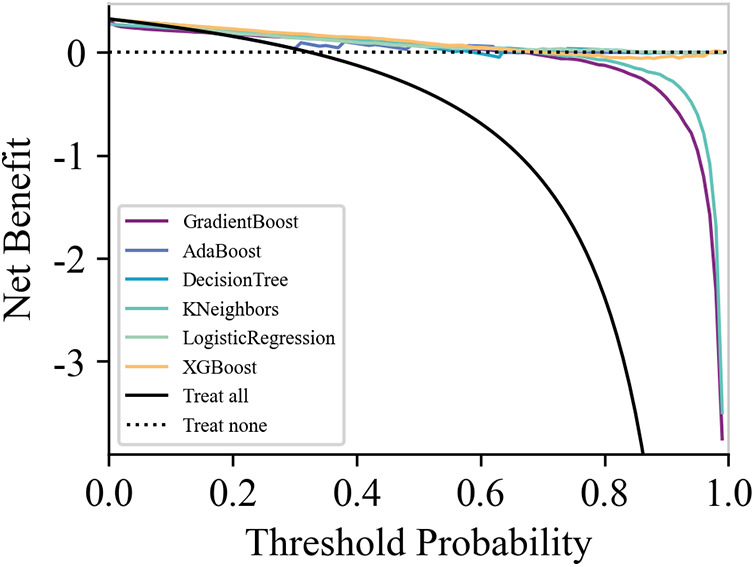
Decision curve analysis of six machine learning models. AdaBoost, adaptive boosting; XGBoost, eXtreme gradient boosting.

We used the top 10 features derived from feature importance scoring by random forest algorithm as input factors. We established six ML methods to perform hypertension classification, including GBDT, AdaBoost, DT, KNN, LR, and XGBoost. The XGBoost model outperformed all the other methods in the validation set, with an AUC of 0.8461 (Figure 4). Accuracy, *f*1 score, precision, recall, and Brier score were also computed to further assess the performance of the six models, and the results are shown in Table 4. Calibration evaluation: Calibration curves for all six models are presented in Figure 5. For the optimal XGBoost model, the Hosmer–Lemeshow goodness-of-fit test yielded *χ*^2^ = 13.9074, df = 8, *P* = 0.0842, indicating no statistically significant deviation from ideal calibration. The Brier score was 0.1545, further confirming good predictive accuracy at the individual probability level. Clinical net benefit evaluation: Decision curve analysis for all six models is shown in Figure 6, with “screen all” and “screen none” as reference strategies. The XGBoost model achieved the highest net clinical benefit across most clinically meaningful threshold probability ranges, supporting the practical value of the web-based screening tool. The XGBoost model outperformed all the other algorithms in the aforementioned assessment measures, and thus, it was chosen for further interpretation analysis.

**Table 4:** Classification performance of six machine learning models for hypertension prevalence identification in the validation cohort.

ML model	AUC	Accuracy	*F*1 score	Precision	Recall	Brier score
GBDT	0.7925	0.7489	0.6224	0.6085	0.6370	0.2099
AdaBoost	0.8214	0.7460	0.6741	0.5780	0.8085	0.1977
DT	0.8340	0.7395	0.6751	0.5675	0.8330	0.1659
KNN	0.7773	0.7533	0.6190	0.6211	0.6169	0.1894
LR	0.8381	0.7337	0.6586	0.5644	0.7906	0.1746
XGBoost	0.8461	0.7721	0.6715	0.6314	0.7171	0.1545

ML, machine learning; GBDT, gradient boosting; AdaBoost, adaptive boosting; DT, decision tree; KNN, k-nearest neighbors; LR, logistic regression; XGBoost, eXtreme gradient boosting; AUC, the area under curve.

### 3.4 Model explainability

**Figure 7:**
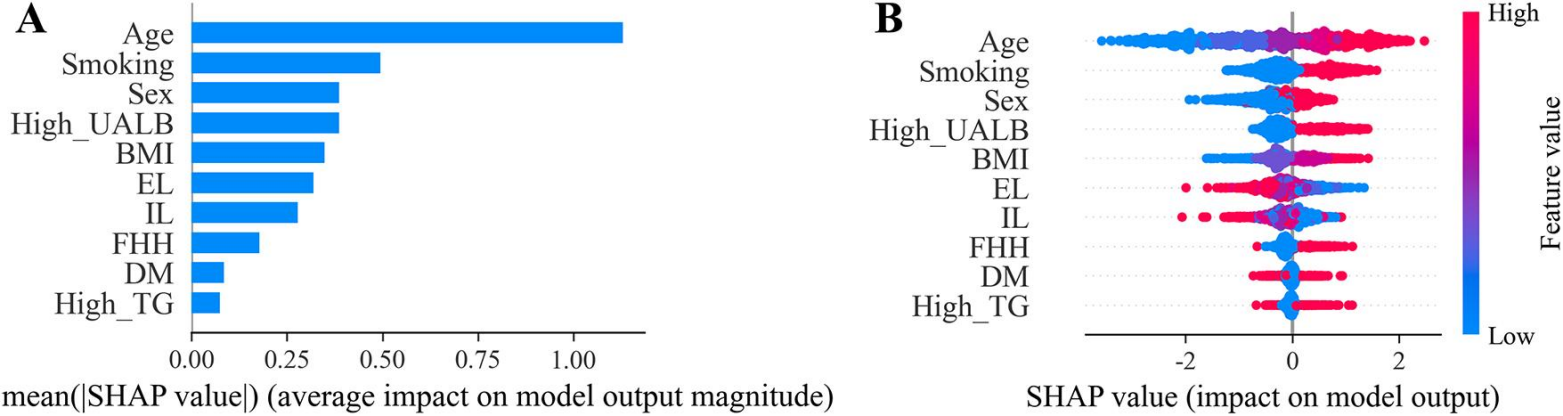
SHAP summary plot of all features that contribute to the XGBoost model. **(A)** Importance ranking of features represented by SHAP. The bar plot shows the importance of each feature in the model prediction output. **(B)** The distribution of the effect of each feature on the model output. Each row represents a feature, and in each row, each dot represents a participant. The color of the dots represents the feature values: red dots represent higher feature values and blue dots represent lower feature values. EL, educational level; IL, income level; BMI, body mass index; DM, diabetes mellitus; FHH, family history of hypertension; TG, triglyceride; UALB, urinary albumin.

Using SHAP values, we were able to unravel how the XGBoost model classifies concurrent hypertension status. The SHAP summary plot presents the feature importance ranking of the XGBoost model, illustrating the contribution magnitude of each variable (Figure 7A). Additionally, we described how certain factors impact the results of the XGBoost classification model using SHAP dependency analysis (Figure 7B). Age, smoking, UALB, BMI, sex, diabetes, TG and family history of hypertension were associated with increased risk of hypertension, whereas educational level and income level were negatively associated with risk of hypertension.

**Figure 8:**
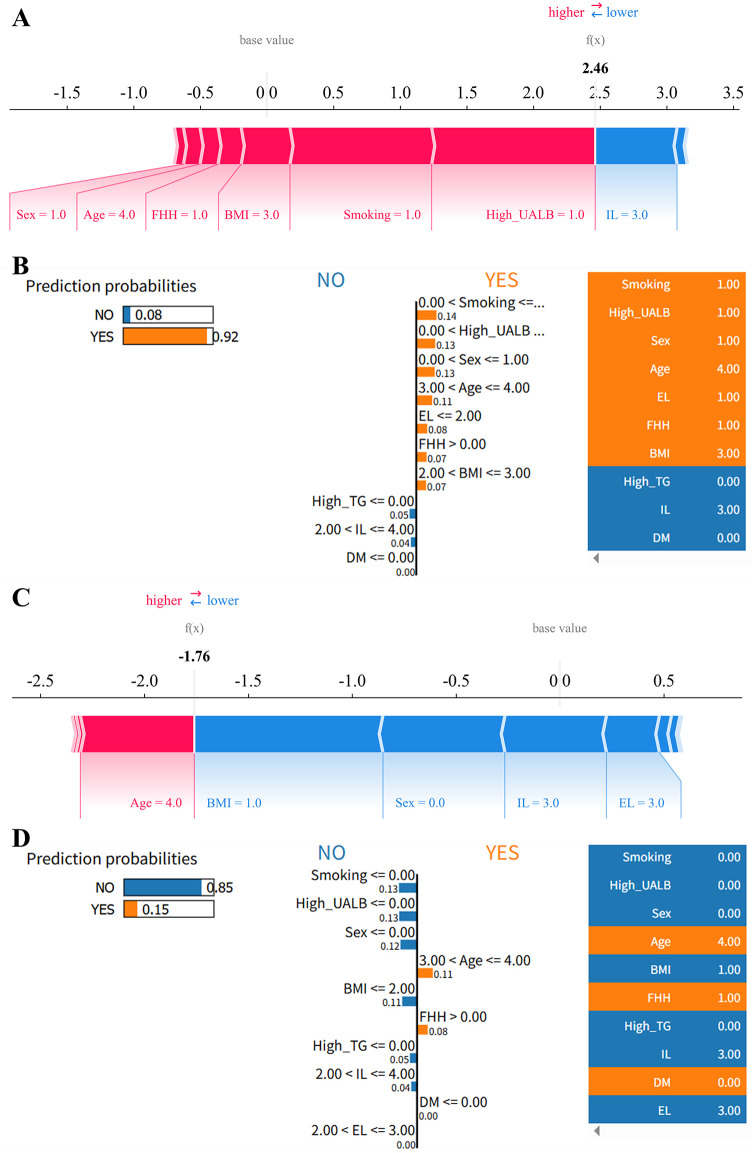
SHAP force analysis and LIME algorithm used to explain individualized predictions. **(A,B)** Were a sample with hypertension explained using SHAP force analysis and LIME algorithm respectively. **(C,D)** Were a sample without hypertension explained using SHAP force analysis and LIME algorithm respectively. In **(A)** and **(C)**, the red and blue bars represent risk and protective factors, respectively, and the longer bars indicate greater feature importance. In **(B)** and **(D)**, the left part displays the classification probability results. The middle part of the figure showed the ranking of all features in terms of the magnitude of their influence on the prediction results, while the length of each feature bar indicated the importance of that feature in the prediction, with longer lengths representing greater importance. The right part lists the specific values of all included features. SHAP, SHapley additive explanation; LIME, local interpretable model-agnostic explanations.

Using two samples from the validation set, we interpreted individualized estimates of hypertension prevalence using SHAP force analysis and the LIME algorithm. The XGBoost model predicted a 92% probability of having prevalent hypertension in the first sample, which was consistent with the actual diagnosis results. The interpretation of the classification results based on SHAP and LIME are depicted in Figures 8A,B. Both analytic approaches consistently demonstrated that smoking, high UALB, male sex, advanced age, low educational level, positive family history of hypertension, and higher body mass index (BMI) were the key drivers pushing the estimated risk upward. In contrast, normal triglyceride level, middle income level, and absence of diabetes mellitus acted as protective factors that lowered the predicted risk. The second sample was predicted to have a 15% probability of having prevalent hypertension but hypertension was not classified as present for this sample. SHAP and LIME results showed that non-smoking, normal UALB, female sex and normal BMI were the most important protective factors, while advanced age was the main risk-increasing factor in this sample (Figures 8C,D).

**Figure 9:**
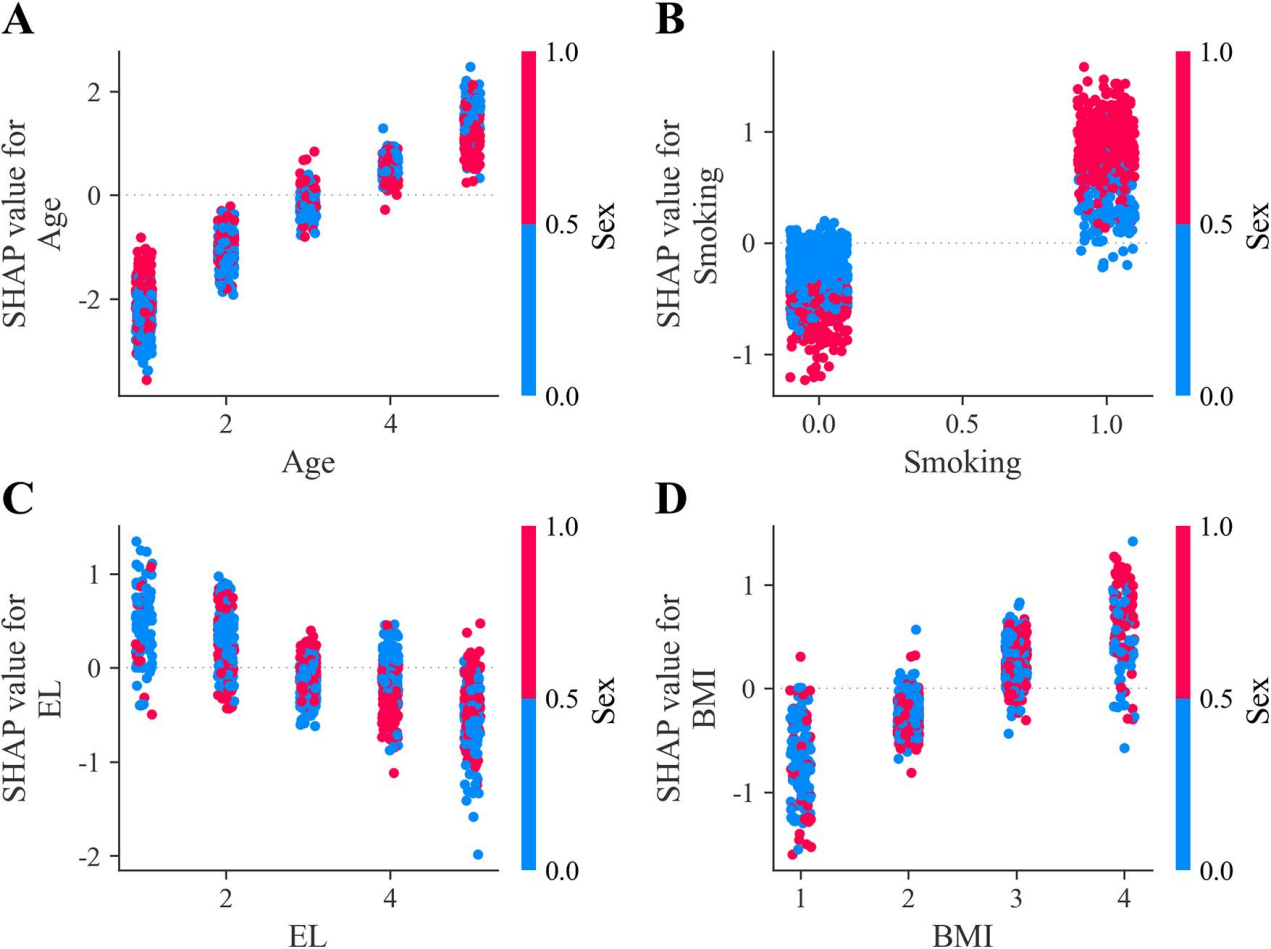
SHAP dependence plot of the interaction between sex and important features. **(A)** The interaction of age and sex. **(B)** The interaction of smoking and sex. **(C)** The interaction of education level and sex. **(D)** The interaction of BMI and sex. The *X*-axis represents the value of the variable labeled on the horizontal axis, and the left *Y*-axis indicates the corresponding SHAP value, reflecting the feature's contribution to model classification outcomes. The color of each point reflected the feature value of the right *Y*-axis heading. When the *x*-coordinate value of the sample point was larger, the variable of the *x*-axis was larger. When the value of the left *Y*-axis coordinate of the sample point was larger, the risk of disease of the sample point was larger. When the color of the sample point was more red, the higher the value of the right *Y*-axis indicator. SHAP, SHapley additive explanation; BMI, body mass index.

In addition, we analyzed the SHAP interaction values to explore the interaction of sex with some predictors (Figures 9A–D). As can be seen in Figure 9A, the risk of hypertension varied by sex, with a progressively higher risk in older females, which was more pronounced at >60 years. The predicted effect of smoking on prevalent hypertension by sex was highest in males (Figure 9B). The interaction effects of educational level, BMI, and sex are shown in Figures 9C,D, demonstrating that the influence of sex was different for different educational levels and different BMI respectively.

### 3.5 Development of an online web page

**Figure 10:**
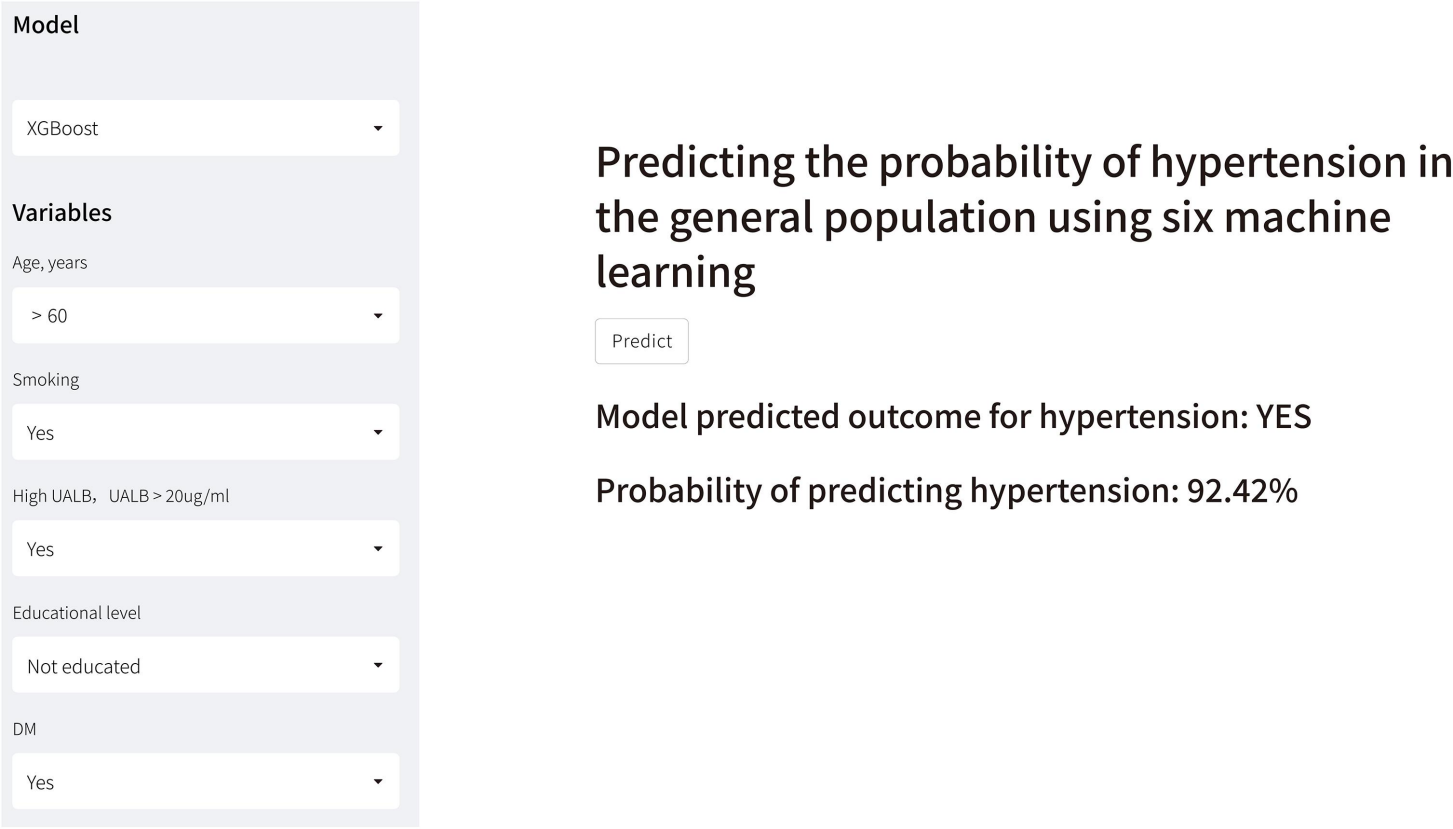
Web-based screening tool for hypertension prevalence identification in the general population.

An online web page was constructed based on six machine learning models, which could be used by clinicians and related researchers to estimate an individual's probability of concurrent prevalent hypertension, with the 10 different features as input data. Notably, the web interface requires users to enter pre-binned categorical variables stratified by the exact same cut-off criteria applied during model development, rather than raw continuous clinical measurements, to ensure full consistency with the model training pipeline and improve research reproducibility. As shown in Figure 10, selecting the XGBoost model in the classification web page and entering information about a representative patient quickly yielded a probability of 93.89% that this patient had hypertension (https://predicting-probability-hypertension-6-uru6.streamlit.app/).

## 4 Discussion

Hypertension has become a growing global public health problem, with increasing prevalence every year (27). Therefore, there is a need for better techniques and methods to analyze the risk factors for this disease in the general population, as well as identify high-risk groups that may benefit from targeted interventions. To estimate the risk of hypertension in the general population, we developed and validated six machine learning techniques utilizing 10 clinical variables. Among them, the XGBoost model showed the best discriminatory power and accuracy performance. Additionally, feature significance and the impact of certain composite substructures on XGBoost classification outputs were demonstrated with SHAP values. Moreover individualized classification was achieved using the LIME algorithm.

The main factors influencing the XGBoost model were age, smoking, UALB, BMI, sex, and family history of hypertension. In this study, age was the most critical risk factor for having prevalent hypertension in the general population. This result is consistent with several studies, which found a positive correlation between age and the risk of having hypertension, and a significantly higher likelihood of hypertension in advanced age (28, 29). This suggests the need to target the middle-aged and elderly population with health promotion interventions to increase its awareness of hypertension and promote regular blood pressure monitoring. Several previous studies have confirmed the differences in the prevalence of hypertension by sex (30, 31). In this research, we discovered that males had a considerably greater prevalence of hypertension than females in the age range under 60. While differences in physiological hormone secretion between males and females may account for this disproportion, males generally have unhealthier lifestyles, such as smoking and alcohol consumption, than females, which are important modifiable risk factors (32–34). In contrast, females over the age of 60 showed a greater risk of hypertension than males. This may be linked to postmenopausal sex hormone changes in females and the expression of genetic susceptibility genes that trigger hypertension after menopause (35, 36). Therefore, the female population over 60 years of age needs to be more active in monitoring blood pressure levels.

Our study found that smoking and UALB were strongly associated with the prevalence of hypertension. As the majority of smokers were males, their risk of hypertension was higher compared with females. In addition, the interaction between these two risk variables may make hypertension more likely. Meanwhile, several studies have shown that non-smokers exposed to secondhand smoke from smokers have a higher chance of having hypertension (37, 38). Therefore, giving up smoking lowers the probability of both the individual and family members having hypertension. Urine microalbumin is an indicator of early renal damage in hypertensive disease, and people with abnormal urine microalbumin levels should be actively screened for hypertension (39). Notably, microalbuminuria is essentially a downstream marker of renal target organ damage secondary to long-standing hypertension, rather than an upstream causal risk factor for incident hypertension. Inclusion of UALB improves the sensitivity of identifying undiagnosed prevalent hypertension and aligns with the community screening positioning of this study; however, the model cannot be used as a prospective tool for predicting future hypertension onset. This clinical distinction must be clearly emphasized to avoid over-interpretation of the model's preventive value. The results of a Mendelian randomization study showed that BMI was an apparent causal risk factor for hypertension and that overweight and obese adults had a greater chance of having hypertension than those with normal BMI, which agrees with this study's findings (40). Maintaining a healthy weight level should be actively promoted as an intervention to prevent and treat hypertension. In addition, similar to the results of existing studies, people with a family history of hypertension have a higher risk of having the disease compared with those without a family history (41). This suggests that hypertension has significant familial aggregation, and active education is needed to monitor blood pressure and control related risk factors in people with a family history of hypertension.

Accurately identifying high-risk people and providing them with prompt management is necessary to lower the prevalence of hypertension in the general population. The advent of machine learning offers a new approach to accurate hypertension classification at population level. Machine learning is a method in the field of artificial intelligence that uses statistics and data mining techniques to train models with large amounts of data to achieve functions such as classification, clustering and pattern recognition of data (42). In the medical field, by analyzing clinical data of a large number of patients using machine learning models, doctors can predict the diagnosis and prognosis of diseases with higher accuracy, and thus provide better prevention and treatment plans based on the classification results (43). Previously, several studies have attempted to model the prevalence of hypertension in the general population using machine learning methods with good classification results. For example, in 2018, Byeong and colleagues developed hypertension classification models based on logistic regression, naive Bayes, and decision trees (44). Among them, the logistic regression classification model had the largest AUC value, with an AUC of 0.700 for men and 0.845 for women. Furthermore, in 2021, an Indian study constructed a classification model for early detection of hypertension risk in a resource-limited setting (45). In the test set, the RF model showed the best performance with an AUC of 0.792. Recently, several modeling studies on hypertension classification have found that XGBoost models perform better than LR, DT, and RF models (46–48). However, all these machine learning models are built based on a limited number of algorithmic tools that need adequate explanation on how they work before they can be used in clinical practice. Here, we built multiple machine learning techniques, including GBDT, AdaBoost, DT, KNN, LR, and XGBoost, and chose XGBoost as it outperformed all others in discriminative power and accuracy. Due to its good classification performance, minimal overfitting, low model complexity, and quick computing benefits, XGBoost is commonly used in clinical practice to construct classification models (49). This method, however, has certain drawbacks. The algorithm's inappropriate usage of hyperparameters may have a significant impact on the training duration and performance of XGBoost models. Additionally, this algorithm is challenging to see and comprehend, which somewhat restricts its application in real-world clinical settings (50). Therefore, we used SHAP values and LIME algorithm to identify and explain the important factors affecting the classification results of the XGBoost model, visualized each component of the classification model, and demonstrated their different contributions to the final results, thus greatly improving the explainability of the model. Finally, to improve usability, we developed an online web page based on the results of this study, where users can enter corresponding information to obtain the probability of having prevalent hypertension.

This research has certain limitations. First, since this research was a cross-sectional survey, the diagnosis of hypertension and the existence of related risk factors could not be ascertained in chronological order. Therefore, the model only classifies concurrent prevalent hypertension at the survey time point and cannot be used to predict future incident hypertension or evaluate long-term onset risk. Second, some survey respondents withdrew from the survey for personal reasons resulting in missing information, which may have led to some selection bias. Third, as the data were based on self-reports, the study was prone to memory bias and reporting bias, with the exception of blood and urine chemistry, anthropometric measures, and blood pressure readings. Fourth, all model development and evaluation were conducted only within a single Hainan cohort via internal train-validation splitting, with no independent external cohort validation. The generalizability of the model to populations with different demographic, dietary and metabolic characteristics remains unconfirmed. Multi-center external validation across multiple independent cohorts is required before widespread clinical deployment of the screening tool. Fifth, urinary microalbumin, as a downstream marker of hypertensive renal target organ damage, may slightly inflate the model's discriminative performance. The model is positioned as a cross-sectional prevalence screening tool and cannot be interpreted as a prospective risk prediction instrument. Sixth, all continuous variables were discretized for clinical practicality, which may cause partial loss of fine-grained continuous information. A head-to-head comparative analysis with native continuous variables was not performed in the current study, and the exact magnitude of performance impact remains to be verified in further research.

## 5 Conclusions

We developed and validated ML models based on clinical characteristics with superior performance in identifying prevalent hypertension in the general population. Applying SHAP values and the LIME algorithm in ML could help identify people at high risk of hypertension for appropriate and timely intervention. At the same time, an online web page based on the research results was developed to support clinical application of the classification model.

## Data Availability

The original contributions presented in the study are included in the article/Supplementary Material, further inquiries can be directed to the corresponding authors.
